# Caspase Exploitation by *Legionella pneumophila*

**DOI:** 10.3389/fmicb.2016.00515

**Published:** 2016-04-13

**Authors:** Kathrin Krause, Amal O. Amer

**Affiliations:** Department of Microbial Infection and Immunity, The Ohio State UniversityColumbus, OH, USA

**Keywords:** caspases, *Legionella pneumophila*, cell death mechanisms, phagosomal maturation, NOD like receptors

## Abstract

*Legionella pneumophila* remains a major health concern, especially for hospitalized patients. *L. pneumophila* in the environment can survive extracellular or as protozoan parasite within amoeba. After human infection it efficiently replicates in alveolar macrophages without activating inflammasome assembly and cleavage of caspase-1. In contrast murine macrophages actively recognize intracellular *L. pneumophila* via inflammasome components which initiate pro-inflammatory cytokine secretion, phagosomal maturation and pyroptotic cell death thereby leading to bacterial restriction. During this process flagellin-dependent and -independent signaling pathways trigger the canonical as well as the non-canonical inflammasome. This review describes the current knowledge about *L. pneumophila*-induced inflammasome pathways in permissive and restrictive host cells.

## Pathogenesis of *Legionella* Species

Gram-negative bacteria of the genus *Legionella* are commonly known as the causative agent of Legionnaires’ disease which account for up to 15% of community-acquired pneumonias (CAP) (Mulazimoglu and Yu, 2001). Twenty species have been shown to cause infections in humans but *Legionella pneumophila* serogroup 1 is responsible for the majority of cases. The first outbreak of Legionnaires’ disease occurred 1976 during the 56th Annual American Legion Convention which is why the subsequently identified etiologic agent was named *Legionella* (McDade et al., 1977). Since the clinical manifestation is an atypical pneumonia with potentially fatal outcome immediate antibiotic treatment is required. Nevertheless, the mortality rate according to the Center of Disease Control (CDC) varies from 5 to 30% with generally higher rates recorded in hospital-aquired cases (Bartram, 2007; Tai et al., 2012). The risk of disease development increases with age, preexisting conditions like chronic lung diseases, cancer and compromised immune defense after organ transplantation, glucocorticoid treatment, or chemotherapy. Although the incidence of Legionnaire’s disease worldwide is unknown, with up to 18.000 annual cases in the USA, increasing numbers have been reported to the CDC in recent years. This might be due to better surveillance and diagnostic resources but also because of improved life expectancy of high-risk patients. In addition to Legionnaires’ disease, *Legionella* species can also cause the non-pneumonic Pontiac fever. This self-limiting, influenza-like infection affects only the upper respiratory tract but is characterized by high attack rates (Tai et al., 2012).

*Legionellae* are globally distributed and predominantly found in natural or human-manufactured aquatic habitats where they live as endosymbionts within free-living amoebae which provide transportation and protection against chemical disinfectants (Abu Kwaik et al., 1998; Richards et al., 2013). Infection usually occurs via inhalation of aerosols or aspiration of water from contaminated sources like showers, air conditioning systems, whirlpools, fountains, or respiratory care devices like air-humidifiers and nebulizers (Cunha et al., 2016). Within the alveolar spaces *L. pneumophila* predominantly replicates in alveolar macrophages and circulating monocytes (Horwitz and Silverstein, 1980). Upon phagocytosis, the bacteria immediately escape the endocytic pathway by avoiding phagosome-lysosome-fusion (Horwitz, 1983b) and phagosomal acidification (Horwitz and Maxfield, 1984) thereby creating a niche for their survival called the *Legionella-*containing vacuole (LCV) (Horwitz, 1983a). Manipulation of host cell trafficking events leads to the encircling of the LCV with host organelles and Endoplasmatic Reticulum (ER) derived vesicles as well as the recruitment of vesicle-transport regulating proteins like Rab1 (Tilney et al., 2001), Sec22b (Derre and Isberg, 2004), and ARF (ADP-ribosylation factor-1) (Kagan and Roy, 2002). The attachment of ER-derived vesicles to the *L. pneumophila*-containing phagosome is a substantial step for LCV-maturation and precedes bacterial replication (Horwitz, 1983a). Beyond that, efficient formation of a replicative compartment relies on the functionality of the Dot/Icm type IV secretion system (T4SS) (Roy et al., 1998). *Legionella* species possess two subtypes of T4SSs related to bacterial conjugation systems: T4ASSs like Lvh, Trb-1 and Trb-2 are not well understood and do not seem to play a role for survival in eukaryotic cells. In contrast, the highly conserved Dot/Icm T4BSS is indispensable for an intracellular life cycle and was shown to affect phagocytosis, LCV biogenesis and replication (Marra et al., 1992; Hilbi et al., 2001). Notably, allows the release of about 300 putative effector proteins with high functional redundancy between individual substrates (Burstein et al., 2009; Schroeder et al., 2010; Zhu et al., 2011; Lifshitz et al., 2013). For instance, multiple effectors have been identified which modulate the function of Rho GTPases thereby manipulating phagosomal trafficking (Hubber and Roy, 2010). Correspondingly, a *dotA* mutant strain fails to escape from the lysosomal degradation pathway, instead, its containing vacuole is characterized by the recruitment of the late endosomal marker proteins Rab7 and LAMP-1 (Roy et al., 1998). Aside from the well described Dot/Icm T4SS *L. pneumophila* strains express a type I (Lss) and type II (Lsp) secretion system. The T2SS, shown to execute the release of various degradative enzymes, also promotes intracellular replication in protozoan hosts and human macrophages whereas the T1SS has no documented role in host–pathogen-interactions (Jacobi and Heuner, 2003; Rossier et al., 2004). Interestingly, in contrast to human macrophages, macrophages from most imbred mouse strains are restrictive to *Legionella* species (Yamamoto et al., 1987, 1988). Further studies revealed that *L. pneumophila* causes efficient inflammasome assembly and caspase-1 activation in murine but not human infections (Ren et al., 2006; Santic et al., 2007; Akhter et al., 2009). Understanding of how *Legionella* avoids detection by innate immune defense mechanisms in permissive macrophages will be enormously relevant to control infections in the future.

## Caspase-1

The importance of caspase-1 as part of the innate immune system has been reported in the context of various types of intracellular infections like *Salmonella enterica* serovar typhimurium, *S. flexneri*, *L. monocytogenes*, *B. pseudomallei*, and *L. pneumophila* (Hilbi et al., 1998; Hersh et al., 1999; Tsuji et al., 2004; Sun et al., 2005). Caspases in general are intracellular cysteine proteases which induce different types of host cell death. Inflammatory caspase-1 becomes activated in multiprotein complexes, called ‘inflammasomes’, which assemble in response to pathogen- or danger-associated molecular patterns (PAMPs or DAMPs) within the cytosol of myeloid cells. Detection of these patterns by cytosolic NOD-like receptors (NLRs) is accompanied by conformational changes leading to oligomerization and recruitment of caspase-1. The subsequent cleavage of caspase-1 is the beginning of an inflammatory cascade with multiple outcomes including cytokine secretion, pore formation resulting in osmotic cell lysis, unconventional protein secretion, modulation of lipid biogenesis as well as gene transcription. Caspase-1 activity upon *L. pneumophila* infection of murine macrophages was first shown to be regulated by the NLR Naip5 (NLR family, apoptosis inhibitory protein 5) (Zamboni et al., 2006).

### Naip5, Nlrc4, and ASC

**FIGURE 1:**
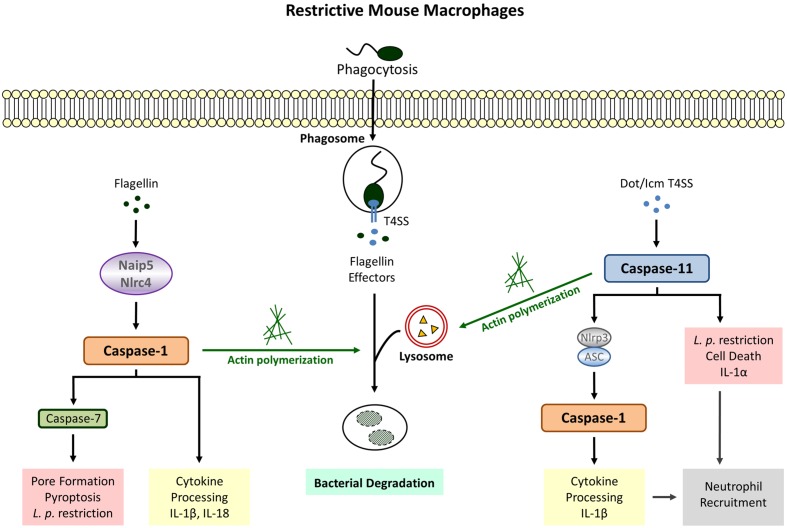
***Legionella pneumophila*-induced Inflammasome Pathways in restrictive macrophages.** Detection of cytosolic flagellin by Naip5/Nlrc4 (canonical inflammasome) leads to cleavage of caspase-1, cytokine release, pyroptosis and bacterial growth restriction. Flagellin-independent recognition of *L. pneumophila* involves the activation of caspase-1 downstream of caspase-11 (non-canonical inflammasome) as well as caspase-1-independent defense strategies executed by caspase-11. Both caspase-1 and caspase-11 promote phagosome-lysosome fusion via modulation of the actin cytoskeleton.

Although mice are generally non-permissive to *L. pneumophila*, macrophages from A/J mice allow bacterial replication (Yamamoto et al., 1988; Yoshida et al., 1991) leading to the discovery that restriction is associated with the Lgn1 locus on chromosome 13 (Beckers et al., 1995). This region harbors multiple copies of Birc1/Naip genes among which Naip5 is underrepresented in the permissive A/J mouse strain and also contains sequence differences at 14 positions (Growney and Dietrich, 2000). Accordingly the expression of Naip5, usually induced after phagocytic events, is significantly decreased in A/J macrophages (Diez et al., 2000) and transgenic restoration of Naip5 in permissive macrophages improves bactericidal activity against *L. pneumophila* (Diez et al., 2003; Wright et al., 2003). Caspase-1 was revealed as the responsible effector molecule for the Naip5-induced antibacterial activity since translocation of bacterial flagellin from the LCV to the host cytosol is detected by Naip5 and leads to cleavage of caspase-1 (Zamboni et al., 2006). Thus, expression of the flagellum-building subunit flagellin in combination with the Dot/Icm type 4 secretion system (T4SS) needed for perforation of the LCV membrane promotes an early caspase-1-mediated cell death leading to reduced bacterial replication and dissemination, whereas macrophages infected with non-flagellated *L. pneumophila* exhibit robust intracellular growth with unimpaired cell viability (Amer et al., 2006; Ren et al., 2006; Akamine et al., 2007). This type of caspase-1-induced cell death, later designated as pyroptosis, is accompanied by the release of the inflammatory cytokines Interleukin (IL) -1β and IL-18 which are directly processed by active caspase-1 (**Figure 1**).

**FIGURE 2:**
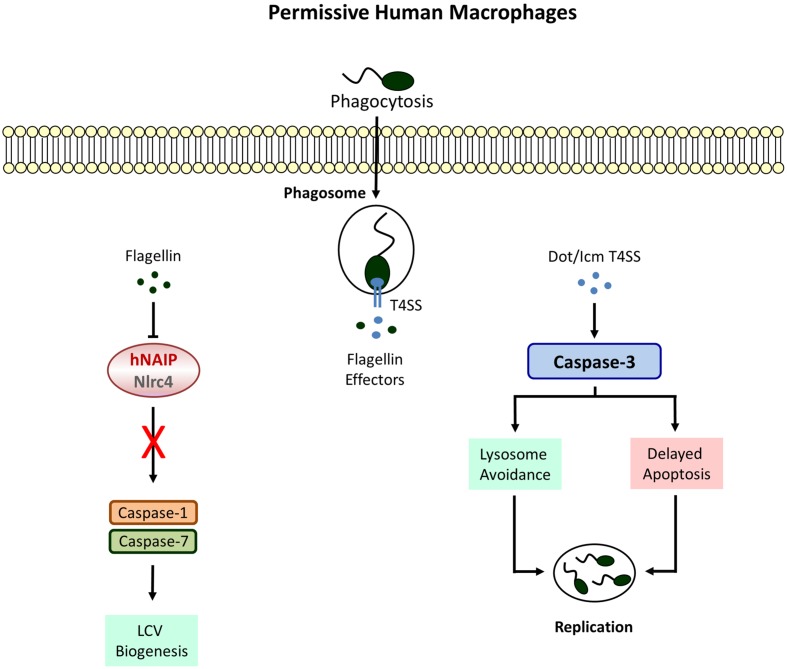
**Defective Inflammasome signaling in permissive macrophages.** Human cell completely lack caspase-1 and -7 activities which allow the formation of a replicative niche called the *Legionella*-containing vacuole (LCV). Phagosome-lysosome fusion is avoided by the activation of caspase-3 leading to bacterial replication and egress.

In addition to Naip5, inflammasome assembly in response *L. pneumophila* has also been demonstrated to require the NLR Nlrc4/Ipaf, another sensor for cytosolic flagellin known to recruit procaspase-1 molecules via homophilic protein-protein interactions within its CARD domain (Amer et al., 2006; Zamboni et al., 2006; Lightfield et al., 2008; Silveira and Zamboni, 2010). Consequently, Nlrc4 deletion abrogates caspase-1 processing; and bactericidal activity, pore formation as well as IL-1β secretion. Furthermore, the Nlrc4-mediated restriction of *L. pneumophila* is entirely dependent on the expression of bacterial flagellin and corresponding mutants exhibit increased replication in bone marrow-derived macrophages and the lungs of infected mice (Pereira et al., 2011b). Since bacterial clearance after recognition of flagellin via Nlrc4 also applies for other non-pneumophila *Legionella* species it is likely to be a general mechanism of host protection against infections caused by *Legionella* ssp. (Silveira and Zamboni, 2010; Pereira et al., 2011a). Interestingly, human macrophages which fail to elicit an activation of caspase-1 in response to *L. pneumophila* infection (**Figure 2**) also possess significantly decreased expression levels of Nlrc4 and the adaptor protein ASC (apoptosis-associated speck-like protein) (Abdelaziz et al., 2011). Although ASC could be ruled out to play a role in Nlrc4/caspase-1-dependent growth restriction of *Legionella* (Zamboni et al., 2006; Pereira et al., 2011a) it was shown to function in flagellin-independent caspase-1-mediated cytokine release (Zamboni et al., 2006; Case et al., 2009). Therefore, it was assumed that *L. pneumophila* activates two distinct caspase-1 inflammasomes in murine macrophages: one responding to flagellin that involves Naip5/Nlrc4 and leads to destruction of the host cell membrane, cell lysis, and bacterial restriction by terminating the replication cycle, and another one dependent on ASC which provokes cytokine production in a flagellin-independent manner. Moreover, ASC has been demonstrated to change the subcellular localization of Nlrc4 and caspase-1 upon infection via recruitment to discrete puncta structures which is important not for the activation but for the cleavage of caspase-1 (Case and Roy, 2011). Even though the redistribution of Nlrc4 is unnecessary for the induction of pyroptosis, ASC takes part in restraining the extent of pore formation in order to maintain cellular homeostasis. These data support the hypothesis of two inflammasomes with distinct locations and functions. Yet, both inflammasome platforms are not completely independent and mutual interference can occur.

After the discovery of caspase-1 deficient mice being accidentally caspase-1/caspase-11 double knockout, the importance of caspase-1 for restriction of *L. pneumophila* in murine infections had to be reevaluated (Kayagaki et al., 2011). Nevertheless, new studies using single caspase-1 knockout macrophages confirmed caspase-1 as responsible molecule for Naip5/Nlrc4-mediated pyroptotic cell death and clearance of flagellated bacteria (Caution et al., 2015; Cerqueira et al., 2015).

### Modulation of the Cytoskeleton

Caspase-1 activity in response to Naip5/Nlrc4 stimulation was also shown to initiate the acquisition of lysosomal instead of ER proteins to the LCV thereby increasing bacterial destruction within phagolysosomal compartments (Amer et al., 2006; Fortier et al., 2007). Recent data provide evidence that caspase-1 targets the actin-binding protein cofilin via activating the phosphatase slingshot (Caution et al., 2015). Dephosporylation represents the active state of cofilin allowing actin severing and polymerization in order to provide a network for vesicular trafficking. Therefore, increased lysosomal degradation of *L. pneumophila* via modulation of the cytoskeleton by caspase-1 might be an attempt to control the intracellular infection before initiation of host cell death (**Figure 1**).

### AIM2 Inflammasome

The recognition of flagellin by the Naip5/Nlrc4 inflammasome appears to be crucial for restriction of *L. pneumophila*, yet there is evidence that bacterial DNA leaking from the LCV can also trigger the AIM2 inflammasome leading to caspase-1 activation and pyroptosis as well (Ge et al., 2012). This process could be observed in human and murine macrophages and was significantly intensified after genetic mutation of the Dot/Icm effector protein SdhA which in the wild-type is important for maintaining the integrity of the vacuolar membrane thereby preventing a sufficient host response (Creasey and Isberg, 2012). Hence, it would be interesting to discover if pharmacological targeting of the LCV might be a way to increase caspase-1 activity in permissive macrophages due to stimulation of the AIM2 inflammasome.

### Negative Regulation of Inflammasome Activity

Research so far has proven that caspase-1 is particularly important to overcome an infection with *L. pneumophila*. Nevertheless, it must be noted that excessive activation of caspase-1 may have detrimental implications for the host. An interesting study from von Moltke et al. (2012) describes the production of eicosanoids as consequence of Naip5/Nlrc4 inflammasome activation with *L. pneumophila*-derived flagellin. Local elevated eicosanoid concentrations are considered to be beneficial by the authors because they promote an influx of immune cells to the side of infection by increasing vascular permeability. In a systemic manner, however, strong eicosanoid generation leads to a substantial loss of vascular fluids, severe inflammation and rapid death. Limitation of inflammasome-dependent signaling in response to *L. pneumophila* was shown to require the ubiquitin-binding protein sequestosome1 (SQSTM1/p62) also involved in autophagy (Ohtsuka et al., 2014). Caspase-1 activity as well as the secretion of IL-1β and IL-18 are significantly enhanced in SQSTM1 deficient macrophages infected with *L. pneumophila*. Additionally, *L. pneumophila*-induced pulmonary inflammation is more severe in the absence of SQSTM1. These results clearly indicate that even though caspase-1 activity is indispensable for establishing a host defense toward *Legionella* species, inflammasome activation needs to be controlled to avoid an overwhelming and self-destructing immune response.

## Caspase-11

Caspase-11 takes part in the non-canonical inflammasome activation pathway in response to various gram-negative bacteria which leads to Nlrp3-dependent processing of caspase-1 (Kayagaki et al., 2011). Caspase-11 as well as the human orthologs caspase-4 and -5 directly bind to cytosolic lipopolysaccharide released from intracellular bacteria and correspondingly, LPS priming has been demonstrated to increase caspase-11 expression which is very low in resting cells (Lin et al., 2000; Hur et al., 2001; Hagar et al., 2013; Shi et al., 2014). Although the activation of caspase-11 as innate immune response against intracellular pathogens is important to control bacteria escaping from the phagosome and replicating in the cytosol of macrophages (Aachoui et al., 2013), the role of caspase-11 regarding vacuolar bacteria remains poorly defined. For instance, caspase-11 deficient mice are susceptible to *L. pneumophila* Lp02 (Akhter et al., 2012). Nevertheless, recent data suggest that caspase-11 is not required for restriction of opsonized *L. pneumophila* JR32 (Cerqueira et al., 2015). The variation in these studies might originate from the difference in strains and growth conditions used, since comparative experiments between JR32 and Lp01, the progenitor of Lp02, displayed diminished intracellular survival of Lp01 in macrophages (Samrakandi et al., 2002). In addition, Lp01 infection stimulates more phagolysosomal fusion events. It is also plausible that opsonization directs the bacterium to a caspase-11-independent trafficking route, whereas bare *Legionella* follows a caspase-11-governed path. Thus, phenotypic difference or enhanced phagocytosis due to opsonization could very well affect the impact of caspase-11-dependent effector functions against *Legionella* ssp.

Although caspase-11 activation in response to *L. pneumophila* was shown to be Nlrc4-independent (Akhter et al., 2012), there is evidence that caspase-11 contributes to IL-1β secretion via triggering Nlrp3/ASC-dependent inflammasome assembly leading to caspase-1 processing (Casson et al., 2013). Interestingly, stimulation of this non-canonical pathway does not require detection of bacterial flagellin but is fully dependent on a functional Dot/Icm T4SS. Similar results could be obtained with caspase-4 in human macrophages (Casson et al., 2015). These data clearly demonstrate that there are other factors aside from flagellin which initiate a host response in *L. pneumophila* infected cells. Furthermore, and provided that disruption of the phagosome by bacterial secretion systems allows the translocation of bacterial components to the cytosol of the host cell, this proves that caspase-11 is activated not only in response to cytosolic but also membrane enclosed vacuolar bacteria (**Figure 1**).

In addition to IL-1β secretion, *L. pneumophila*-stimulated caspase-11 induction also participates in an Nlrp3/ASC- and Naip5/Nlrc4-independent release of IL-1α associated with cell death and completely dependent on the presence of the T4SS (Case et al., 2013; Casson et al., 2013). Correspondingly, downregulation of caspase-4 in *L. pneumophila*-infected human monocyte-derived macrophages (MDMs) significantly reduces cytotoxicity and IL-1α production (Casson et al., 2015). IL-1α has been demonstrated to be the primary initiator for IL-1 receptor (IL-1R)-mediated neutrophil recruitment (Barry et al., 2013) and reduction of pulmonary neutrophil infiltration revealed increased mortality during *L. pneumophila* infection (Tateda et al., 2001a,b). However, defective neutrophil recruitment and bacterial replication in IL-1α knockout mice do not reach the same level as observed in IL-1R^-/-^ mice indicating that IL-1β also plays a role (Barry et al., 2013). Another study using neutralizing antibodies confirmed a dominant role for IL-1α in *L. pneumophila*-induced neutrophil recruitment but only depletion of both cytokines recapitulated the IL-1R^-/-^ phenotype (Casson et al., 2013). Therefore, IL-1β clearly participates in the recruitment of neutrophils upon *L. pneumophila* infection. Given that flagellin is dispensable for both caspase-11-dependent cell death and IL-1α/β secretion, caspase-11 activation appears to be an autonomous pathway for detection of *Legionella* ssp. to maintain host defense against bacteria evading the canonical inflammasome (Case et al., 2013).

Like caspase-1, caspase-11 also facilitates endocytic fusion events in response to *L. pneumophila* by modulating the actin cytoskeleton (Akhter et al., 2012). Caspase-11 deficient macrophages fail to form F-actin structures around the LCV resulting in decreased co-localization of *L. pneumophila* with lysosomal compartments. Earlier studies demonstrated that caspase-11 interacts with Aip1 (actin-interacting protein 1) in order to promote cofilin-mediated actin polymerization (Li et al., 2007). With regard to intracellular *L. pneumophila* a physical interaction between caspase-11 and actin itself could be demonstrated in murine macrophages (Akhter et al., 2012). Furthermore, *L. pneumophila*-induced expression of the lipid-raft associated membrane protein flotillin-1, known to accumulate on mature phagosomes (Dermine et al., 2001), is severely diminished in caspase-11 deficient macrophages (Akhter et al., 2012). Thus, regulation of actin dynamics by caspase-11 appears to be a crucial defense mechanism toward *L. pneumophila* (**Figure 1**).

## Other Caspases

Macrophages permissive to *L. pneumophila* do not display caspase-1 activity (Santic et al., 2007; Abdelaziz et al., 2011). Instead a strong Dot/Icm T4SS-dependent activation of caspase-3 could be demonstrated in these cells (Gao and Abu Kwaik, 1999; Zink et al., 2002). However, this does not lead to an immediate apoptotic response. In fact *L. pneumophila* causes rather a remarkable resistance toward cell death inducing agents at early stages of infection (Molmeret et al., 2004; Abu-Zant et al., 2005). The upregulation of anti-apoptotic genes due to nuclear translocation of the transcription factor NFκB is partially responsible for preventing host cell destruction (Losick and Isberg, 2006; Abu-Zant et al., 2007). Furthermore, SidF, a phosphatidylinositol phosphatase, blocks the apoptotic pathway by neutralizing the pro-apoptotic Bcl-2 family members BNIP3 and Bcl-rambo in U937 cells (Banga et al., 2007). The effector protein SdhA, known to protect the integrity of the LCV, is crucial for the intracellular survival of *L. pneumophila* and an *sdhA* mutant strain fails to avert host cell death (Laguna et al., 2006). Although caspase-3 has no particular role in caspase-1 competent primary murine macrophages (Zamboni et al., 2006), pre-activation of caspase-3 was shown to improve bacterial clearance considerably in human U937 and murine J774 macrophage cell lines (Abu-Zant et al., 2005). Thus, it appears that caspase-3 activation is a distinct host response, independent of caspase-1 which is actively antagonized by intracellular *L. pneumophila*. This hypothesis is supported by the fact that murine dendritic cells (DCs) induce an early caspase-3 mediated apoptotic cell death upon *L. pneumophila* infection to limit bacterial replication and dissemination (Nogueira et al., 2009). Interestingly and despite the functionality of the Naip5/caspase-1 pathway, pyroptosis is not needed to efficiently eradicate *L. pneumophila* from DCs whereas deletion of apoptotic activators like Bax and Bak results in enhanced bacterial proliferation. With regard to *L. pneumophila* five Dot/Icm T4SS proteins have been identified to contribute to caspase-3 activation in DCs among which the phospholipase VipD provokes cytochrome c release via mitochondrial destabilization (Zhu et al., 2013). Accordingly, the ability to induce apoptosis is compromised in a corresponding mutant strain and accompanied by improved bacterial replication. Surprisingly, there is no reduction of *L. pneumophila* numbers neither in human nor in murine macrophages after loss of these proteins.

Instead of restraining intracellular replication, caspase-3 activity rather promotes growth of *L. pneumophila* in U937 macrophages due to cleavage of the Rab5 effector Rabaptin-5 thereby enabling evasion from the endosomal-lysosomal degradation pathway (Molmeret et al., 2004). Moreover, after pharmacological inhibition of caspase-3 increased phagosomal maturation and trafficking to lysosomes could be observed which results in impaired bacterial survival. Yet, the importance of caspase-3 was proven to be limited to the bacterial internalization process to subvert vesicle biogenesis whereas inhibiting caspase-3 activity at later stages of infection remains ineffective. Further studies are certainly needed to clarify why rapid stimulation of the mitochondrial apoptosis pathway is successful in DCs leading to *L. pneumophila* restriction but not in permissive macrophages where (regardless of active caspase-3) the apoptotic response is severely delayed and considered to serve as a tool for bacterial dissemination.

Restrictive macrophages activate caspase-1 in response to *L. pneumophila* but the question was raised if there are further substrates downstream of caspase-1 which take part in the *Legionella*-confining phenotype. Intriguingly, caspase-7, so far known to participate only in the classic apoptotic cell death pathway, was identified to be processed by caspase-1 (Lamkanfi et al., 2008). Caspase-1-dependent activation of caspase-7 in murine C57BL/6 macrophages upon infection with *L. pneumophila* requires detection of bacterial flagellin by the host proteins Naip5 and Nlrc4 (Akhter et al., 2009; Kofoed and Vance, 2011). Moreover, caspase-7 activity is responsible for restraining bacterial replication: firstly, because of promoting vesicle trafficking leading to bacterial degradation within phagolysosomes. Secondly, caspase-7 was shown to contribute to early apoptosis which eliminates the replicative habitat (Akhter et al., 2009). Despite the ability of permissive A/J macrophages to activate caspase-1 they lack caspase-7 activity. This might account for their incapacity to control *L. pneumophila* infections. Furthermore, pre-activation of caspase-3 was shown to reduce bacterial loads in permissive human and murine cells but the substance staurosporine used in these experiments is a general activator of apoptosis which also affects caspase-7 (Abu-Zant et al., 2007). Fluorescent substrates measuring caspase-3 activity are known to recognize caspase-7 as well. Additional investigations are needed to clarify whether caspase-7 plays a role in human cells and if caspase-3 and -7 have distinct or overlapping functions.

## NOD-Like Receptors Related Functions

The primary function of inflammasome complexes so far is considered to translate cytosolic danger signals recognized by NLR to activate downstream caspases which then initiate effector functions. Yet, there is increasing evidence that NLRs alone are capable of mediating defense mechanisms against intracellular pathogens. With regard *to L. pneumophila*, the fact that permissive A/J macrophages harboring a mutant Naip5 protein display similar levels of active caspase-1 like restrictive C57BL/6 macrophages in response to cytosolic flagellin suggests that Naip5 has other functions aside from merely sensing bacterial presence (Lamkanfi et al., 2007). Consistently, transgenic C57BL/6 macrophages carrying the A/J Naip5 protein allow growth of the *L. pneumophila* wild-type. Similar results were found for Nlrc4 deficient macrophages which display higher replication of *Legionella* ssp. than single caspase-1 as well as caspase-1/11 double knockout macrophages indicating the existence of a caspase-1-independent host response triggered by Nlrc4 (Pereira et al., 2011b; Cerqueira et al., 2015). Experiments with human lung epithelial cells not expressing Nlrc4 also revealed an increase in intracellular bacterial numbers after downregulation of human NAIP known to share 68% homology with murine Naip5, whereas overexpression of both exogenous Nlrc4 and endogenous NAIP improved bacterial clearance (Vinzing et al., 2008). Enhanced bacterial loads after knockdown of NAIP or Nlrc4 were also found in primary human alveolar and MDMs (Vinzing et al., 2008). Human macrophages lack caspase-1 activity even in the presence of both molecules which emphasizes the idea of caspase-1-independent functions for NLRs. Moreover, caspase-1 is dispensable for the recruitment of Nlrc4 to subcellular puncta structures in murine macrophages (Case and Roy, 2011) and Nlrc4 also interacts with Caspase-11 (Akhter et al., 2012), thus other inflammasome platforms might exist. Overexpression of hNAIP in murine RAW264.7 macrophages successfully promotes caspase-1 activation and bacterial growth restriction in response to flagellin-competent *L. pneumophila* (Katagiri et al., 2012). Contradictorily, hNAIP expressed in human-derived HEK293 cells does not respond to *L. pneumophila* flagellin (Zhao et al., 2011). The opposite results in human and mouse remains unclear and requires further research.

## Unresolved Aspects

Inflammasome assembly in response to *L. pneumophila* is very complex with multiple interaction possibilities between individual proteins. Although caspase-11 has been shown to be dispensable for the Nlrc4-mediated activation of caspase-1 (Akhter et al., 2012; Cerqueira et al., 2015), there is evidence that caspase-11 binds components of the flagellin-induced Nlrc4 inflammasome. Independently of caspase-1, caspase-11 plays a role for detection of flagellin-deficient *Legionella* ssp. as well as IL-1α secretion thereby facilitating neutrophil recruitment to the side of infection. Thus, both caspases are capable of either complementing each other or initiating completely separate defense strategies. Bacterial virulence proteins, however, or potential sensor proteins upstream of caspase-11 leading to its activation are not sufficiently clarified. Guanylate-binding proteins promote caspase-11 dependent pyroptosis in IFN-γ stimulated macrophages due to recognition of *L. pneumophila*-derived cytosolic LPS (Pilla et al., 2014). Additional studies in this field may provide a better understanding of the molecular mechanism behind *L. pneumophila*-induced caspase-11 activation.

It is not surprising that bacteria develop mechanisms to actively suppress or evade inflammasome activation. Biofilms in fresh water structures are the main source of infection. Interestingly, biofilm-derived bacteria do not express flagellin and for this reason neither initiate caspase-1/-7 activation nor pyroptosis in murine macrophages (Abu Khweek et al., 2013). Nevertheless, in human cells the absence of active caspase-1 was proven to be flagellin-independent which raises the question whether *L. pneumophila* might inhibit the function of Nlrc4 with a yet unknown factor or if human Nlrc4 *per se* is non-responsive toward *Legionella*-derived flagellin.

Finally, permissive macrophages still exhibit attributes of programmed cell death but the purpose of these caspase-1/11-independent pathways is controversial (Tao et al., 2013). Overall, it becomes obvious that host cell death in response to *Legionella* species is a delicate process which can be elicited to manage and contain intracellular infections but also provides an escape mechanism for bacterial dissemination leading to severe inflammation. Understanding how *Legionella* overcomes host defense mechanism in human cells will be important to develop new therapeutic strategies.

## Author Contributions

KK wrote the manuscript and made the figures. AA edited the manuscript and the figures.

## Conflict of Interest Statement

The authors declare that the research was conducted in the absence of any commercial or financial relationships that could be construed as a potential conflict of interest.

## References

[B1] Aachoui Y. Leaf I. A. Hagar J. A. Fontana M. F. Campos C. G. Zak D. E. (2013). Caspase-11 protects against bacteria that escape the vacuole. *Science* 339 975–978. 10.1126/science.1230751 23348507 PMC3697099

[B2] Abdelaziz D. H. Gavrilin M. A. Akhter A. Caution K. Kotrange S. Khweek A. A. (2011). Apoptosis-associated speck-like protein (ASC) controls *Legionella pneumophila* infection in human monocytes. *J. Biol. Chem.* 286 3203–3208. 10.1074/jbc.M110.197681 21097506 PMC3030324

[B3] Abu Khweek A. Fernandez Davila N. S. Caution K. Akhter A. Abdulrahman B. A. Tazi M. (2013). Biofilm-derived *Legionella pneumophila* evades the innate immune response in macrophages. *Front. Cell Infect Microbiol.* 3:18 10.3389/fcimb.2013.00018 PMC3664316 23750338

[B4] Abu Kwaik Y. Gao L. Y. Stone B. J. Venkataraman C. Harb O. S. (1998). Invasion of protozoa by *Legionella pneumophila* and its role in bacterial ecology and pathogenesis. *Appl. Environ. Microbiol.* 64 3127–3133. 9726849 10.1128/aem.64.9.3127-3133.1998 PMC106699

[B5] Abu-Zant A. Jones S. Asare R. Suttles J. Price C. Graham J. (2007). Anti-apoptotic signalling by the Dot/Icm secretion system of *L. pneumophila*. *Cell Microbiol.* 9 246–264. 10.1111/j.1462-5822.2006.00785.x 16911566

[B6] Abu-Zant A. Santic M. Molmeret M. Jones S. Helbig J. Abu Kwaik Y. (2005). Incomplete activation of macrophage apoptosis during intracellular replication of *Legionella pneumophila*. *Infect. Immun.* 73 5339–5349. 10.1128/IAI.73.9.5339-5349.2005 16113249 PMC1231138

[B7] Akamine M. Higa F. Haranaga S. Tateyama M. Mori N. Heuner K. (2007). Interferon-gamma reverses the evasion of Birc1e/Naip5 gene mediated murine macrophage immunity by *Legionella pneumophila* mutant lacking flagellin. *Microbiol. Immunol.* 51 279–287. 10.1111/j.1348-0421.2007.tb03909.x 17380047

[B8] Akhter A. Caution K. Abu Khweek A. Tazi M. Abdulrahman B. A. Abdelaziz D. H. (2012). Caspase-11 promotes the fusion of phagosomes harboring pathogenic bacteria with lysosomes by modulating actin polymerization. *Immunity* 37 35–47. 10.1016/j.immuni.2012.05.001 22658523 PMC3408798

[B9] Akhter A. Gavrilin M. A. Frantz L. Washington S. Ditty C. Limoli D. (2009). Caspase-7 activation by the Nlrc4/Ipaf inflammasome restricts *Legionella pneumophila* infection. *PLoS Pathog.* 5:e1000361 10.1371/journal.ppat.1000361 PMC2657210 19343209

[B10] Amer A. Franchi L. Kanneganti T. D. Body-Malapel M. Ozoren N. Brady G. (2006). Regulation of *Legionella phagosome* maturation and infection through flagellin and host Ipaf. *J. Biol. Chem.* 281 35217–35223. 10.1074/jbc.M604933200 16984919

[B11] Banga S. Gao P. Shen X. Fiscus V. Zong W. X. Chen L. (2007). *Legionella pneumophila* inhibits macrophage apoptosis by targeting pro-death members of the Bcl2 protein family. *Proc. Natl. Acad. Sci. U.S.A.* 104 5121–5126. 10.1073/pnas.0611030104 17360363 PMC1829273

[B12] Barry K. C. Fontana M. F. Portman J. L. Dugan A. S. Vance R. E. (2013). IL-1alpha signaling initiates the inflammatory response to virulent *Legionella pneumophila* in vivo. *J. Immunol.* 190 6329–6339. 10.4049/jimmunol.1300100 23686480 PMC3682686

[B13] Bartram J. (2007). *Legionella and the Prevention of Legionellosis.* Geneva: World Health Organization.

[B14] Beckers M. C. Yoshida S. Morgan K. Skamene E. Gros P. (1995). Natural resistance to infection with *Legionella pneumophila*: chromosomal localization of the Lgn1 susceptibility gene. *Mamm. Genome* 6 540–545. 10.1007/BF00356173 8589525

[B15] Burstein D. Zusman T. Degtyar E. Viner R. Segal G. Pupko T. (2009). Genome-scale identification of *Legionella pneumophila* effectors using a machine learning approach. *PLoS Pathog* 5:e1000508 10.1371/journal.ppat.1000508 PMC2701608 19593377

[B16] Case C. L. Kohler L. J. Lima J. B. Strowig T. De Zoete M. R. Flavell R. A. (2013). Caspase-11 stimulates rapid flagellin-independent pyroptosis in response to *Legionella pneumophila*. *Proc. Natl. Acad. Sci. U.S.A.* 110 1851–1856. 10.1073/pnas.1211521110 23307811 PMC3562791

[B17] Case C. L. Roy C. R. (2011). Asc modulates the function of NLRC4 in response to infection of macrophages by *Legionella pneumophila*. *MBio* 2:e00117 10.1128/mBio.00117-11 PMC3269931 21771913

[B18] Case C. L. Shin S. Roy C. R. (2009). Asc and Ipaf Inflammasomes direct distinct pathways for caspase-1 activation in response to *Legionella pneumophila*. *Infect. Immun.* 77 1981–1991. 10.1128/IAI.01382-08 19237518 PMC2681768

[B19] Casson C. N. Copenhaver A. M. Zwack E. E. Nguyen H. T. Strowig T. Javdan B. (2013). Caspase-11 activation in response to bacterial secretion systems that access the host cytosol. *PLoS Pathog.* 9:e1003400 10.1371/journal.ppat.1003400 PMC3675167 23762026

[B20] Casson C. N. Yu J. Reyes V. M. Taschuk F. O. Yadav A. Copenhaver A. M. (2015). Human caspase-4 mediates noncanonical inflammasome activation against gram-negative bacterial pathogens. *Proc. Natl. Acad. Sci. U.S.A.* 112 6688–6693. 10.1073/pnas.1421699112 25964352 PMC4450384

[B21] Caution K. Gavrilin M. A. Tazi M. Kanneganti A. Layman D. Hoque S. (2015). Caspase-11 and caspase-1 differentially modulate actin polymerization via RhoA and Slingshot proteins to promote bacterial clearance. *Sci. Rep.* 5:18479 10.1038/srep18479 PMC4685268 26686473

[B22] Cerqueira D. M. Pereira M. S. Silva A. L. Cunha L. D. Zamboni D. S. (2015). Caspase-1 but Not Caspase-11 Is Required for NLRC4-mediated pyroptosis and restriction of infection by flagellated legionella species in mouse macrophages and in vivo. *J. Immunol.* 195 2303–2311. 10.4049/jimmunol.1501223 26232428

[B23] Creasey E. A. Isberg R. R. (2012). The protein SdhA maintains the integrity of the Legionella-containing vacuole. *Proc. Natl. Acad. Sci. U.S.A.* 109 3481–3486. 10.1073/pnas.1121286109 22308473 PMC3295292

[B24] Cunha B. A. Burillo A. Bouza E. (2016). Legionnaires’ disease. *Lancet* 387 376–385. 10.1016/S0140-6736(15)60078-2 26231463

[B25] Dermine J. F. Duclos S. Garin J. St-Louis F. Rea S. Parton R. G. (2001). Flotillin-1-enriched lipid raft domains accumulate on maturing phagosomes. *J. Biol. Chem.* 276 18507–18512. 10.1074/jbc.M101113200 11279173

[B26] Derre I. Isberg R. R. (2004). *Legionella pneumophila* replication vacuole formation involves rapid recruitment of proteins of the early secretory system. *Infect. Immun.* 72 3048–3053. 10.1128/IAI.72.5.3048-3053.2004 15102819 PMC387905

[B27] Diez E. Lee S. H. Gauthier S. Yaraghi Z. Tremblay M. Vidal S. (2003). Birc1e is the gene within the Lgn1 locus associated with resistance to *Legionella pneumophila*. *Nat. Genet.* 33 55–60. 10.1038/ng1065 12483212

[B28] Diez E. Yaraghi Z. Mackenzie A. Gros P. (2000). The neuronal apoptosis inhibitory protein (Naip) is expressed in macrophages and is modulated after phagocytosis and during intracellular infection with *Legionella pneumophila*. *J. Immunol.* 164 1470–1477. 10.4049/jimmunol.164.3.1470 10640764

[B29] Fortier A. De Chastellier C. Balor S. Gros P. (2007). Birc1e/Naip5 rapidly antagonizes modulation of phagosome maturation by *Legionella pneumophila*. *Cell Microbiol.* 9 910–923. 10.1111/j.1462-5822.2006.00839.x 17087731

[B30] Gao L. Y. Abu Kwaik Y. (1999). Activation of caspase 3 during *Legionella pneumophila*-induced apoptosis. *Infect. Immun.* 67 4886–4894. 10456945 10.1128/iai.67.9.4886-4894.1999 PMC96823

[B31] Ge J. Gong Y. N. Xu Y. Shao F. (2012). Preventing bacterial DNA release and absent in melanoma 2 inflammasome activation by a *Legionella effector* functioning in membrane trafficking. *Proc. Natl. Acad. Sci. U.S.A.* 109 6193–6198. 10.1073/pnas.1117490109 22474394 PMC3341053

[B32] Growney J. D. Dietrich W. F. (2000). High-resolution genetic and physical map of the Lgn1 interval in C57BL/6J implicates Naip2 or Naip5 in *Legionella pneumophila* pathogenesis. *Genome Res.* 10 1158–1171. 10.1101/gr.10.8.1158 10958634 PMC310929

[B33] Hagar J. A. Powell D. A. Aachoui Y. Ernst R. K. Miao E. A. (2013). Cytoplasmic LPS activates caspase-11: implications in TLR4-independent endotoxic shock. *Science* 341 1250–1253. 10.1126/science.1240988 24031018 PMC3931427

[B34] Hersh D. Monack D. M. Smith M. R. Ghori N. Falkow S. Zychlinsky A. (1999). The *Salmonella invasin* SipB induces macrophage apoptosis by binding to caspase-1. *Proc. Natl. Acad. Sci. U.S.A.* 96 2396–2401. 10.1073/pnas.96.5.2396 10051653 PMC26795

[B35] Hilbi H. Moss J. E. Hersh D. Chen Y. Arondel J. Banerjee S. (1998). *Shigella*-induced apoptosis is dependent on caspase-1 which binds to IpaB. *J. Biol. Chem.* 273 32895–32900. 10.1074/jbc.273.49.32895 9830039

[B36] Hilbi H. Segal G. Shuman H. A. (2001). Icm/dot-dependent upregulation of phagocytosis by *Legionella pneumophila*. *Mol. Microbiol.* 42 603–617. 10.1046/j.1365-2958.2001.02645.x 11722729

[B37] Horwitz M. A. (1983a). Formation of a novel phagosome by the Legionnaires’ disease bacterium (*Legionella pneumophila*) in human monocytes. *J. Exp. Med.* 158 1319–1331. 10.1084/jem.158.4.1319 6619736 PMC2187375

[B38] Horwitz M. A. (1983b). The Legionnaires’ disease bacterium (*Legionella pneumophila*) inhibits phagosome-lysosome fusion in human monocytes. *J. Exp. Med.* 158 2108–2126. 6644240 10.1084/jem.158.6.2108 PMC2187157

[B39] Horwitz M. A. Maxfield F. R. (1984). *Legionella pneumophila* inhibits acidification of its phagosome in human monocytes. *J. Cell Biol.* 99 1936–1943. 10.1083/jcb.99.6.1936 6501409 PMC2113576

[B40] Horwitz M. A. Silverstein S. C. (1980). Legionnaires’ disease bacterium (*Legionella pneumophila*) multiples intracellularly in human monocytes. *J. Clin. Invest.* 66 441–450. 10.1172/JCI109874 7190579 PMC371671

[B41] Hubber A. Roy C. R. (2010). Modulation of host cell function by *Legionella pneumophila* type IV effectors. *Annu. Rev. Cell Dev. Biol.* 26 261–283. 10.1146/annurev-cellbio-100109-104034 20929312

[B42] Hur J. Kim S. Y. Kim H. Cha S. Lee M. S. Suk K. (2001). Induction of caspase-11 by inflammatory stimuli in rat astrocytes: lipopolysaccharide induction through p38 mitogen-activated protein kinase pathway. *FEBS Lett.* 507 157–162. 10.1016/S0014-5793(01)02975-1 11684090

[B43] Jacobi S. Heuner K. (2003). Description of a putative type I secretion system in *Legionella pneumophila*. *Int. J. Med. Microbiol.* 293 349–358. 10.1078/1438-4221-00276 14695063

[B44] Kagan J. C. Roy C. R. (2002). Legionella phagosomes intercept vesicular traffic from endoplasmic reticulum exit sites. *Nat. Cell Biol.* 4 945–954. 10.1038/ncb883 12447391

[B45] Katagiri N. Shobuike T. Chang B. Kukita A. Miyamoto H. (2012). The human apoptosis inhibitor NAIP induces pyroptosis in macrophages infected with *Legionella pneumophila*. *Microbes Infect.* 14 1123–1132. 10.1016/j.micinf.2012.03.006 22504023

[B46] Kayagaki N. Warming S. Lamkanfi M. Vande Walle L. Louie S. Dong J. (2011). Non-canonical inflammasome activation targets caspase-11. *Nature* 479 117–121. 10.1038/nature10558 22002608

[B47] Kofoed E. M. Vance R. E. (2011). Innate immune recognition of bacterial ligands by NAIPs determines inflammasome specificity. *Nature* 477 592–595. 10.1038/nature10394 21874021 PMC3184209

[B48] Laguna R. K. Creasey E. A. Li Z. Valtz N. Isberg R. R. (2006). A *Legionella pneumophila*-translocated substrate that is required for growth within macrophages and protection from host cell death. *Proc. Natl. Acad. Sci. U.S.A.* 103 18745–18750. 10.1073/pnas.0609012103 17124169 PMC1656969

[B49] Lamkanfi M. Amer A. Kanneganti T. D. Munoz-Planillo R. Chen G. Vandenabeele P. (2007). The Nod-like receptor family member Naip5/Birc1e restricts *Legionella pneumophila* growth independently of caspase-1 activation. *J. Immunol.* 178 8022–8027. 10.4049/jimmunol.178.12.8022 17548639

[B50] Lamkanfi M. Kanneganti T. D. Van Damme P. Vanden Berghe T. Vanoverberghe I. Vandekerckhove J. (2008). Targeted peptidecentric proteomics reveals caspase-7 as a substrate of the caspase-1 inflammasomes. *Mol. Cell. Proteomics* 7 2350–2363. 10.1074/mcp.M800132-MCP200 18667412 PMC2596343

[B51] Li J. Brieher W. M. Scimone M. L. Kang S. J. Zhu H. Yin H. (2007). Caspase-11 regulates cell migration by promoting Aip1-Cofilin-mediated actin depolymerization. *Nat. Cell Biol.* 9 276–286. 10.1038/ncb1541 17293856

[B52] Lifshitz Z. Burstein D. Peeri M. Zusman T. Schwartz K. Shuman H. A. (2013). Computational modeling and experimental validation of the Legionella and Coxiella virulence-related type-IVB secretion signal. *Proc. Natl. Acad. Sci. U.S.A.* 110 E707–E715. 10.1073/pnas.1215278110 23382224 PMC3581968

[B53] Lightfield K. L. Persson J. Brubaker S. W. Witte C. E. Von Moltke J. Dunipace E. A. (2008). Critical function for Naip5 in inflammasome activation by a conserved carboxy-terminal domain of flagellin. *Nat. Immunol.* 9 1171–1178. 10.1038/ni.1646 18724372 PMC2614210

[B54] Lin X. Y. Choi M. S. Porter A. G. (2000). Expression analysis of the human caspase-1 subfamily reveals specific regulation of the CASP5 gene by lipopolysaccharide and interferon-gamma. *J. Biol. Chem.* 275 39920–39926. 10.1074/jbc.M007255200 10986288

[B55] Losick V. P. Isberg R. R. (2006). NF-kappaB translocation prevents host cell death after low-dose challenge by *Legionella pneumophila*. *J. Exp. Med.* 203 2177–2189. 10.1084/jem.20060766 16940169 PMC2118400

[B56] Marra A. Blander S. J. Horwitz M. A. Shuman H. A. (1992). Identification of a *Legionella pneumophila* locus required for intracellular multiplication in human macrophages. *Proc. Natl. Acad. Sci. U.S.A.* 89 9607–9611. 10.1073/pnas.89.20.9607 1409673 PMC50181

[B57] McDade J. E. Shepard C. C. Fraser D. W. Tsai T. R. Redus M. A. Dowdle W. R. (1977). Legionnaires’ disease: isolation of a bacterium and demonstration of its role in other respiratory disease. *N. Engl. J. Med.* 297 1197–1203. 10.1056/NEJM197712012972202 335245

[B58] Molmeret M. Zink S. D. Han L. Abu-Zant A. Asari R. Bitar D. M. (2004). Activation of caspase-3 by the Dot/Icm virulence system is essential for arrested biogenesis of the Legionella-containing phagosome. *Cell Microbiol.* 6 33–48. 10.1046/j.1462-5822.2003.00335.x 14678329

[B59] Mulazimoglu L. Yu V. L. (2001). Can Legionnaires disease be diagnosed by clinical criteria? A critical review. *Chest* 120 1049–1053. 11591534 10.1378/chest.120.4.1049

[B60] Nogueira C. V. Lindsten T. Jamieson A. M. Case C. L. Shin S. Thompson C. B. (2009). Rapid pathogen-induced apoptosis: a mechanism used by dendritic cells to limit intracellular replication of *Legionella pneumophila*. *PLoS Pathog.* 5:e1000478 10.1371/journal.ppat.1000478 PMC2689937 19521510

[B61] Ohtsuka S. Ishii Y. Matsuyama M. Ano S. Morishima Y. Yanagawa T. (2014). SQSTM1/p62/A170 regulates the severity of *Legionella pneumophila* pneumonia by modulating inflammasome activity. *Eur. J. Immunol.* 44 1084–1092. 10.1002/eji.201344091 24374573

[B62] Pereira M. S. Marques G. G. Dellama J. E. Zamboni D. S. (2011a). The Nlrc4 Inflammasome Contributes to Restriction of Pulmonary Infection by Flagellated Legionella spp. that Trigger Pyroptosis. *Front. Microbiol.* 2:33 10.3389/fmicb.2011.00033 PMC3109297 21687424

[B63] Pereira M. S. Morgantetti G. F. Massis L. M. Horta C. V. Hori J. I. Zamboni D. S. (2011b). Activation of NLRC4 by flagellated bacteria triggers caspase-1-dependent and -independent responses to restrict *Legionella pneumophila* replication in macrophages and in vivo. *J. Immunol.* 187 6447–6455. 10.4049/jimmunol.1003784 22079982

[B64] Pilla D. M. Hagar J. A. Haldar A. K. Mason A. K. Degrandi D. Pfeffer K. (2014). Guanylate binding proteins promote caspase-11-dependent pyroptosis in response to cytoplasmic LPS. *Proc. Natl. Acad. Sci. U.S.A.* 111 6046–6051. 10.1073/pnas.1321700111 24715728 PMC4000848

[B65] Ren T. Zamboni D. S. Roy C. R. Dietrich W. F. Vance R. E. (2006). Flagellin-deficient Legionella mutants evade caspase-1- and Naip5-mediated macrophage immunity. *PLoS Pathog.* 2:e18 10.1371/journal.ppat.0020018 PMC1401497 16552444

[B66] Richards A. M. Von Dwingelo J. E. Price C. T. Abu Kwaik Y. (2013). Cellular microbiology and molecular ecology of Legionella-amoeba interaction. *Virulence* 4 307–314. 10.4161/viru.24290 23535283 PMC3710333

[B67] Rossier O. Starkenburg S. R. Cianciotto N. P. (2004). *Legionella pneumophila* type II protein secretion promotes virulence in the A/J mouse model of Legionnaires’ disease pneumonia. *Infect. Immun.* 72 310–321. 10.1128/IAI.72.1.310-321.2004 14688110 PMC344012

[B68] Roy C. R. Berger K. H. Isberg R. R. (1998). *Legionella pneumophila* DotA protein is required for early phagosome trafficking decisions that occur within minutes of bacterial uptake. *Mol. Microbiol.* 28 663–674. 10.1046/j.1365-2958.1998.00841.x 9632267

[B69] Samrakandi M. M. Cirillo S. L. Ridenour D. A. Bermudez L. E. Cirillo J. D. (2002). Genetic and phenotypic differences between *Legionella pneumophila* strains. *J. Clin. Microbiol.* 40 1352–1362. 10.1128/JCM.40.4.1352-1362.2002 11923356 PMC140379

[B70] Santic M. Asare R. Doric M. Abu Kwaik Y. (2007). Host-dependent trigger of caspases and apoptosis by *Legionella pneumophila*. *Infect. Immun.* 75 2903–2913. 10.1128/IAI.00147-07 17420236 PMC1932860

[B71] Schroeder G. N. Petty N. K. Mousnier A. Harding C. R. Vogrin A. J. Wee B. (2010). *Legionella pneumophila* strain 130b possesses a unique combination of type IV secretion systems and novel Dot/Icm secretion system effector proteins. *J. Bacteriol.* 192 6001–6016. 10.1128/JB.00778-10 20833813 PMC2976443

[B72] Shi J. Zhao Y. Wang Y. Gao W. Ding J. Li P. (2014). Inflammatory caspases are innate immune receptors for intracellular LPS. *Nature* 514 187–192. 10.1038/nature13683 25119034

[B73] Silveira T. N. Zamboni D. S. (2010). Pore formation triggered by Legionella spp. *is an Nlrc*4 inflammasome-dependent host cell response that precedes pyroptosis. *Infect. Immun.* 78 1403–1413. 10.1128/IAI.00905-09 20048047 PMC2825914

[B74] Sun G. W. Lu J. Pervaiz S. Cao W. P. Gan Y. H. (2005). Caspase-1 dependent macrophage death induced by *Burkholderia pseudomallei*. *Cell Microbiol.* 7 1447–1458. 10.1111/j.1462-5822.2005.00569.x 16153244

[B75] Tai J. Benchekroun M. N. Ennaji M. M. Mekkour M. Cohen N. (2012). Nosocomial Legionnaires’ Disease: risque and prevention. *Front. Sci.* 2:75 10.5923/j.fs.20120204.03

[B76] Tao L. Zhu W. Hu B. J. Qu J. M. Luo Z. Q. (2013). Induction of rapid cell death by an environmental isolate of *Legionella pneumophila* in mouse macrophages. *Infect. Immun.* 81 3077–3088. 10.1128/IAI.00252-13 23753633 PMC3754228

[B77] Tateda K. Moore T. A. Deng J. C. Newstead M. W. Zeng X. Matsukawa A. (2001a). Early recruitment of neutrophils determines subsequent T1/T2 host responses in a murine model of *Legionella pneumophila* pneumonia. *J. Immunol.* 166 3355–3361. 10.4049/jimmunol.166.5.3355 11207291

[B78] Tateda K. Moore T. A. Newstead M. W. Tsai W. C. Zeng X. Deng J. C. (2001b). Chemokine-dependent neutrophil recruitment in a murine model of Legionella pneumonia: potential role of neutrophils as immunoregulatory cells. *Infect. Immun.* 69 2017–2024. 10.1128/IAI.69.4.2017-2024.2001 11254553 PMC98125

[B79] Tilney L. G. Harb O. S. Connelly P. S. Robinson C. G. Roy C. R. (2001). How the parasitic bacterium *Legionella pneumophila* modifies its phagosome and transforms it into rough ER: implications for conversion of plasma membrane to the ER membrane. *J. Cell Sci.* 114 4637–4650. 11792828 10.1242/jcs.114.24.4637

[B80] Tsuji N. M. Tsutsui H. Seki E. Kuida K. Okamura H. Nakanishi K. (2004). Roles of caspase-1 in Listeria infection in mice. *Int. Immunol.* 16 335–343. 10.1093/intimm/dxh041 14734619

[B81] Vinzing M. Eitel J. Lippmann J. Hocke A. C. Zahlten J. Slevogt H. (2008). NAIP and Ipaf control *Legionella pneumophila* replication in human cells. *J. Immunol.* 180 6808–6815. 10.4049/jimmunol.180.10.6808 18453601

[B82] von Moltke J. Trinidad N. J. Moayeri M. Kintzer A. F. Wang S. B. Van Rooijen N. (2012). Rapid induction of inflammatory lipid mediators by the inflammasome in vivo. *Nature* 490 107–111. 10.1038/nature11351 22902502 PMC3465483

[B83] Wright E. K. Goodart S. A. Growney J. D. Hadinoto V. Endrizzi M. G. Long E. M. (2003). Naip5 affects host susceptibility to the intracellular pathogen *Legionella pneumophila*. *Curr. Biol.* 13 27–36. 10.1016/S0960-9822(02)01359-3 12526741

[B84] Yamamoto Y. Klein T. W. Newton C. A. Friedman H. (1988). Interaction of *Legionella pneumophila* with peritoneal macrophages from various mouse strains. *Adv. Exp. Med. Biol.* 239 89–98. 10.1007/978-1-4757-5421-6_10 3264454

[B85] Yamamoto Y. Klein T. W. Newton C. A. Widen R. Friedman H. (1987). Differential growth of *Legionella pneumophila* in guinea pig versus mouse macrophage cultures. *Infect. Immun.* 55 1369–1374. 3570468 10.1128/iai.55.6.1369-1374.1987 PMC260522

[B86] Yoshida S. Goto Y. Mizuguchi Y. Nomoto K. Skamene E. (1991). Genetic control of natural resistance in mouse macrophages regulating intracellular *Legionella pneumophila* multiplication in vitro. *Infect. Immun.* 59 428–432. 1987055 10.1128/iai.59.1.428-432.1991 PMC257758

[B87] Zamboni D. S. Kobayashi K. S. Kohlsdorf T. Ogura Y. Long E. M. Vance R. E. (2006). The Birc1e cytosolic pattern-recognition receptor contributes to the detection and control of *Legionella pneumophila* infection. *Nat. Immunol.* 7 318–325. 10.1038/ni1305 16444259

[B88] Zhao Y. Yang J. Shi J. Gong Y. N. Lu Q. Xu H. (2011). The NLRC4 inflammasome receptors for bacterial flagellin and type III secretion apparatus. *Nature* 477 596–600. 10.1038/nature10510 21918512

[B89] Zhu W. Banga S. Tan Y. Zheng C. Stephenson R. Gately J. (2011). Comprehensive identification of protein substrates of the Dot/Icm type IV transporter of *Legionella pneumophila*. *PLoS ONE* 6:e17638 10.1371/journal.pone.0017638 PMC3052360 21408005

[B90] Zhu W. Hammad L. A. Hsu F. Mao Y. Luo Z. Q. (2013). Induction of caspase 3 activation by multiple *Legionella pneumophila* Dot/Icm substrates. *Cell Microbiol.* 15 1783–1795. 10.1111/cmi.12157 23773455 PMC3797225

[B91] Zink S. D. Pedersen L. Cianciotto N. P. Abu-Kwaik Y. (2002). The Dot/Icm type IV secretion system of *Legionella pneumophila* is essential for the induction of apoptosis in human macrophages. *Infect Immun.* 70 1657–1663. 10.1128/IAI.70.3.1657-1663.2002 11854262 PMC127815

